# Investigating Why and How Young Adults Use Protective Behavioral Strategies for Alcohol and Marijuana Use: Protocol for Developing a Randomized Controlled Trial

**DOI:** 10.2196/37106

**Published:** 2022-04-19

**Authors:** Melissa A Lewis, Dana M Litt, Anne M Fairlie, Jason R Kilmer, Emma Kannard, Raul Resendiz, Travis Walker

**Affiliations:** 1 Health Behavior and Health Systems University of North Texas Health Science Center Fort Worth, TX United States; 2 Psychiatry and Behavioral Sciences University of Washington Seattle, WA United States

**Keywords:** alcohol use, marijuana use, protective behavioral strategies, intervention development, young adults

## Abstract

**Background:**

Young adulthood is associated with increased alcohol and marijuana use compared with other developmental periods. Alcohol and marijuana use place individuals at high risk for acute and long-term negative consequences. Despite the relatively large cross-sectional and longitudinal literature on protective behavioral strategies (PBS; behaviors that individuals can use to limit consequences and reduce substance use), little is known about why young adults choose to use PBS on specific occasions or why they might use PBS differently across occasions (ie, quality and consistency). There is significant room for improvement in the conceptualization, application, and understanding of PBS.

**Objective:**

This study aims to develop a novel, brief web-based and SMS text messaging intervention, with input from young adults who use alcohol and marijuana, which addresses the extent to which motivations for PBS use and nonuse (marijuana or alcohol) and the quality of PBS use (the degree of effectiveness or degree of implementation) differ when using alcohol alone versus concurrently or simultaneously with marijuana.

**Methods:**

This research will be conducted in 2 phases. Phase 1 will involve web-based focus groups (N=100) and cognitive interviews (N=10) to determine why young adults (aged 18-24 years) use or do not use specific PBS related to alcohol and marijuana use and elicit feedback on how motivations and the quality of PBS could be incorporated into a web-based and SMS text messaging PBS intervention as well as elicit feedback on developed intervention material. In phase 2, young adults (N=200; aged 18-24 years), who typically use alcohol and marijuana for at least 2 days per week, will be randomized to either the intervention or waitlist control group. The intervention will be brief, web-based, focusing on self-selected alcohol and marijuana PBS messages and motives for using alcohol- and marijuana-related PBS, and including intervention content delivered via SMS text messages 3 days a week (random day, Friday, and Saturday) over 8 consecutive weeks. All participants will report on PBS use, motivations for PBS use (and nonuse), quality of PBS use, and alcohol and marijuana use in morning surveys timed to occur the day after the intervention SMS text messages for those in the intervention group.

**Results:**

Recruitment and enrollment for phase 1 began in January 2022. Recruitment for phase 2 is anticipated to begin in January 2023. Upon completion of the phase 2 pilot, we will examine the feasibility, acceptability, and preliminary effect sizes of the newly developed brief web-based and SMS text messaging intervention.

**Conclusions:**

This study will provide an in-depth understanding of young adults’ PBS use and has the potential to develop a more efficacious intervention for co-occurring or simultaneous alcohol and marijuana behaviors.

**Trial Registration:**

ClinicalTrials.gov NCT04978129; https://clinicaltrials.gov/ct2/show/NCT04978129

**International Registered Report Identifier (IRRID):**

DERR1-10.2196/37106

## Introduction

### Background

Young adulthood is associated with increased alcohol use compared with other developmental periods. Recent national US estimates show that 62.6% of young adults have consumed alcohol in the year before the data collection [1]. Acute alcohol-related negative consequences, including academic or occupational impairment, blackouts, injury, and death, occur in academic, interpersonal, social, and health domains [2-4]. Moreover, an estimated 29.2% of persons aged 18 to 29 years with past-year alcohol use have an alcohol use disorder [5]. Although a meta-analysis of 6 national surveys indicated that there had not been a significant increase in alcohol consumption among persons aged 18 to 29 years over the past decade [6], young adult alcohol use continues to be a public health concern in the United States because of the risk of acute and long-term negative consequences. The development of more efficacious interventions to reduce the proportion of young adults who engage in excessive alcohol use and experience negative consequences is a key priority of the National Institute on Alcohol Abuse and Alcoholism.

Unlike alcohol use, young adult marijuana use has increased in the past decade [1], whereas the perceived risk of regular marijuana use among adolescents and young adults continues to decrease [1,7]. Among young adults in the United States, the lifetime rate of marijuana use is 60.1%, with rates of past 30-day use at 24.1% and daily use at 8% [1]. Frequent and long-term marijuana use is linked to acute consequences, including decreased cognitive functioning [8], as well as longer-term consequences, including discontinuous college enrollment and unemployment [9,10].

### Risks of Concurrent and Simultaneous Alcohol and Marijuana Use

Most people who use both alcohol and marijuana do so simultaneously [11,12]. Simultaneous alcohol and marijuana (SAM) use is defined as using alcohol and marijuana at the same time so that their effects overlap. Concurrent alcohol and marijuana (CAM) use is often defined in the literature by individuals retrospectively reporting both alcohol and marijuana use within the same period (eg, past month and past year), without reference to experiencing overlapping effects. Almost one-quarter of adolescents and young adults who report alcohol or marijuana use also report SAM use when asked about the past year or the last party attended [13,14]. SAM use, compared with marijuana use alone or CAM use, is associated with an increased risk of consequences [13,15-20]. In this study, all participants will be young adults who use alcohol and marijuana. Given our focus on substance use behavior across days, we will use a more precise definition of CAM use, such that CAM use occurs when individuals report the use of both alcohol and marijuana on the same day but not so that their effects overlap. It is increasingly important that interventions focus on both alcohol and marijuana use and evaluate whether they are effective at reducing CAM and SAM use.

### Protective Behavioral Strategies

#### Alcohol Protective Behavioral Strategies

A way of preventing or reducing risk is promoting the use of protective behavioral strategies (PBS), which are behaviors that individuals can use to limit negative consequences and reduce alcohol use [21]. Several types of alcohol PBS have often been examined; three common types include (1) limiting or stopping drinking (eg, stopping drinking at a predetermined time), (2) manner of drinking (eg, drinking slowly rather than gulp or chug), and (3) serious harm reduction (eg, using a designated driver) [22]. Cross-sectional and longitudinal research demonstrate global associations between PBS and drinking behavior, such that greater overall use of PBS are negatively associated with the quantity and frequency of drinking and its consequences [23,24]. Emerging event-level research, such as our work, has demonstrated that the use of PBS varies across days, similar to the type of PBS used [23,25-29]. Specifically, although the manner of drinking PBS are negatively associated with alcohol use on a given day, event-level research shows that limiting or stopping PBS and the serious harm reduction of PBS are associated with more drinking on a given day. These findings for limiting or stopping PBS and serious harm reduction PBS contrast with research showing a negative global association, where individuals who tend to use more PBS also tend to report less alcohol use overall.

#### Marijuana PBS

As research on marijuana continues to address changing routes of administration, increasing concentration or potency (eg, tetrahydrocannabinol levels), and emerging products, research also acknowledges the challenges with reducing risks associated with marijuana use. Certainly, harm reduction approaches acknowledge that any steps toward reduced risk are steps in the right direction; however, although there are clear guidelines for *low-risk* drinking (eg, provided by the National Institute on Alcohol Abuse and Alcoholism), the same cannot be said of marijuana use. Very little research has been conducted on marijuana PBS. This developing area of research shows that the cross-sectional and longitudinal findings for marijuana PBS (eg, avoiding mixing marijuana with other drugs, avoiding high-frequency use, and using marijuana only among trusted peers) [30,31] are similar to those for alcohol [31-36]. Bravo et al [37] demonstrated that both alcohol and marijuana PBS help explain the association between known risk factors (ie, sex, age at substance use onset, substance use motives, and impulsivity-like traits) and associated consequences among those who report CAM use (used alcohol and marijuana for at least 1 day in the past month). Prince et al [36] examined PBS interventions to reduce young adult marijuana use and found that, regardless of intervention conditions, greater daily PBS use was associated with lower quantities of marijuana use that day, such that using PBS in a given episode was associated with lower marijuana use (ie, approximately half of a standard joint less of marijuana or 0.25 g) compared with episodes when no PBS were used. As this study asked whether PBS were used for each event rather than asking about specific PBS, it was not possible to determine if certain PBS were associated with less use or consequences. Pearson et al [38] found that a marijuana PBS total score was associated with fewer marijuana sessions and a lower subjective high in participants from a daily diary study among college students. Overall, our findings suggest that marijuana PBS use at the daily level needs to be further defined and examined so that marijuana PBS can be effectively targeted in interventions. As the literature is limited, there is significant room for improvement in the conceptualization, application, and understanding of marijuana PBS. This research has the potential to add significantly to the literature as it will allow a fine-grained examination of the efficacy of marijuana PBS on alcohol use, marijuana use, and CAM or SAM use.

#### Event-Level Associations

Research has yet to examine how alcohol and marijuana PBS use on a given day relate to an individual’s use of alcohol or marijuana alone in comparison with CAM or SAM use days. Knowledge in this area is necessary to inform PBS interventions or interventions with a PBS component, aiming to reduce alcohol and marijuana use. Given the concerning CAM and SAM use rates among young adults in the United States, alcohol interventions need to focus on marijuana use and examine whether these interventions can reduce CAM and SAM use, alcohol and marijuana use, and related consequences, as well as increase alcohol and marijuana PBS use. Specifically, an important research question that this study will answer is the extent to which the use of PBS (marijuana or alcohol) or the quality of PBS use differs if using alcohol alone versus concurrently or simultaneously with marijuana and how motivations to use or not use PBS affect these associations. Moreover, little is known regarding how alcohol or marijuana PBS differs on CAM or SAM use days compared with alcohol-only days. Thus, this study will collect event-level data to determine which alcohol or marijuana PBS are effective at reducing use and consequences when CAM or SAM use occurs compared with alcohol use alone.

#### Motivations for Use and Nonuse of PBS (Why Use PBS)

Event-level research that shows differences in PBS use across days suggests that young adults’ motivations for using certain types of PBS may also differ across days [26-29,39]. However, motivations to use PBS for alcohol or marijuana across days have yet to be examined. For example, if a young adult wants to drink heavily but does not experience negative consequences, the individual might use fewer (if any) limiting or stopping PBS and use more serious harm reduction PBS. Very little is currently known about why young adults may or may not use various PBS when consuming alcohol. Given that many brief alcohol interventions for young adults introduce PBS [40], it is important to understand *why* (eliciting personally relevant reasons to make a change and decide to use PBS) young adults may elect to use or not use PBS on specific occasions or use it with more quality (ie, better implementation) across days. There are many reasons why an individual may not use any PBS or a specific PBS (eg, unaware of strategy, embarrassed, do not want to use strategy as they want to drink more and drink longer), and understanding why and when certain strategies are preferred or disregarded could lead to critical refinements for the manner in which PBS are incorporated into alcohol interventions. For example, if an individual is not motivated to use any PBS, the common protocol of increasing the awareness of strategies and providing skills training regarding how to implement PBS may be insufficient to encourage or enable the individual to use PBS [41,42]. Furthermore, it is important to understand the motivations behind *not* using PBS, as these motivations may be barriers to change (ie, implementing PBS and reducing consequences). Research shows that friends can influence PBS use [27], such that greater friends’ use of serious harm reduction PBS was associated with greater serious harm reduction PBS use by the participants. Friends’ use of limiting or stopping and manner of drinking strategies were not associated with participants’ drinking habits, consequences, or PBS. It may be that friends have high a use of or encourage the use of serious harm reduction strategies as a means of drinking more heavily, which suggests that friends’ PBS use may be a barrier to using serious harm reduction PBS to reduce one’s own use or consequences.

Recent research by Bravo et al [43] identified themes for reasons of using PBS (prevention of specific alcohol-related consequences and general safety) and themes for reasons of not using PBS (goal conflict, ineffectiveness, difficulty of implementation, and negative peer or social repercussions). The limitations of this research are that it focused on reasons for use and nonuse of PBS overall rather than for each specific strategy, and participants responded to survey items rather than being involved in focus groups or cognitive interviews, which allowed for a more in-depth examination of motivations, especially at this early stage of creating a foundational scientific knowledge base. The study by Bravo et al [43] also did not examine potential gender differences in PBS use and nonuse. Despite these limitations, the findings suggest that greater specificity is needed regarding why and when PBS are or are not used, so that intervention content can be tailored to include motivations for choosing to use or not use PBS, which has the potential to improve PBS-based interventions. Given that research based on the Health Belief Model has shown that real or perceived barriers prevent individuals from engaging in health-protective behaviors [44-46], it is important to better understand the motivations behind the nonuse of PBS. Interventions may need to focus on reducing perceived barriers or reasons for nonuse of PBS so that young adults can become more aware of how and when they can effectively implement these strategies. Thus, this research will inform our understanding of why PBS use among young adults is low by examining reasons for not using PBS, as well as barriers to implementing PBS, which can ultimately inform refinements to intervention content.

Regarding marijuana-related PBS, Prince et al [35] conducted focus groups with a community sample of young adults and found themes surrounding the reasons for regulating marijuana use (eg, health or legal problems and interpersonal), as well as strategies to moderate marijuana use or reduce the risk of consequences (eg, distraction and existential or spiritual strategies). There is even less literature examining the reasons for use and nonuse of marijuana PBS compared with alcohol PBS, thus highlighting the pressing need for additional research to inform how to best incorporate marijuana PBS into interventions. Research, such as the work described herein, is needed to determine when and why young adults may or may not decide to use strategies to reduce harm when using either alcohol and marijuana alone as well as PBS on days with CAM or SAM use. Thus, this research will allow for potentially more effective interventions and, ultimately, a greater public health impact by incorporating the reasons *why* PBS might be used rather than only listing options for *how* to use PBS.

#### Quality of PBS Use

In addition to the need to examine the reasons for PBS use or nonuse, there is a need to determine how well (ie, the quality with which) young adults implement PBS. For example, 2 young adults may indicate that they both watch their drinks to avoid harm; 1 carries their drink and keeps it with them at all times, and the other periodically looks at their drink on a table in a crowded room. Furthermore, it may be true that the same person engages in PBS with different qualities on different days (eg, on Friday, a person watches their drink while hanging out with a few close friends at dinner in a restaurant, and the same individual on Saturday watches their drink more sporadically while with a larger group of friends in a bar). These examples relate to the quality of alcohol in PBS and can be extended to marijuana PBS. Thus, the quality of implementing both alcohol and marijuana PBS likely varies across individuals and across occasions. The manner in which PBS are currently included in brief interventions (ie, skills training) does not address the varying quality with which PBS may be implemented, which likely results in differing levels of PBS effectiveness. Furthermore, many feedback-based interventions present the number of PBS used by the participant (ie, quantity) with nothing about the actual impact, usefulness, consistency, or effectiveness (ie, quality of implementation). Specifically, it is possible that someone may report using a certain PBS in a manner that is not actually protective against risk (ie, poor-quality PBS use). Therefore, it is important to elucidate the ways in which people use PBS, as well as the extent to which the manner of use is effective and protective, which can then inform brief interventions.

#### Gender Differences in PBS Use

Research is beginning to investigate the motivations for PBS use; however, research has yet to examine the possibility that the motivations and the quality of PBS use may differ by gender (eg, male, female, or nonbinary). For example, female college students may opt to bring their own alcohol to parties (eg, bring your own bottle) to know what is in their drink. However, research suggests that bringing alcohol to parties does not prevent sexual victimization in college [47]. Gender differences such as this are important as interventions may need to highlight or discuss varying motivations for alcohol and marijuana PBS use. Moreover, these findings demonstrate the need to evaluate gender-specific motivations for PBS use across a range of risk behaviors, as alcohol or marijuana PBS could be used to avoid consequences not directly related to substance use.

#### Current Intervention Approaches

PBS are often incorporated into multicomponent brief interventions [48,49] or SMS text message interventions [50,51] in the form of skills training for reducing both alcohol and marijuana use. The PBS are consistent with the harm reduction model, with the idea that any steps toward reduced risk are steps in the right direction [52,53]. Many personalized feedback interventions (PFIs) aim to identify a *hook*, or personally relevant reason to change, by using motivational interviewing principles and strategies that support building young adults’ motivation to change their drinking or marijuana use behavior. Brief interventions that use motivational interviewing emphasize the importance of meeting people where they are in terms of their readiness to change and suggest that if personally relevant reasons for change can be elicited, contemplation of change or commitment to change can result. Interventions that target PBS provide *action stage* suggestions; however, if people are in precontemplation, contemplation, or preparation, there is a disconnect between these action stage suggestions and where they might actually be in terms of their readiness to change their substance use or even their PBS use. Furthermore, the PBS component of PFIs generally occurs at the end of the intervention or when of interest to the participant so that moderation tips and strategies (typically *action stage* strategies) can be provided after reviewing the intervention content (eg, norm comparisons or goals) that is likely to prompt the contemplation of change and increase the individual’s motivation to reduce harm.

Collectively, most intervention components focus on *why* to change alcohol or marijuana use, whereas the PBS component focuses on *how* to change substance use. The investigation of what might be gained by avoiding, restricting, or limiting CAM or SAM use, in particular, has the potential to highlight any unique motivations for concerns or challenges associated with *why* someone would want to make changes in PBS use or CAM or SAM use. For interventions to be the most efficacious, content that can adequately result in the contemplation of change (ie, *why* changes might occur) is necessary to set the stage for PBS implementation (ie, *how* to make those changes). Prevention efforts have the potential to improve if the *why* is also a focus for PBS (why use certain PBS over other PBS in a given context), as examined in this project. By examining how interventions can better elicit personally relevant reasons to make a change in one’s behavior and engage in quality PBS use for alcohol or marijuana use, we have the potential to optimize current intervention approaches.

Despite theoretical support for the inclusion of PBS content in PFIs (65% of interventions do so) [54], findings have been inconclusive regarding whether PBS use mediates college student alcohol intervention efficacy, with only some studies showing evidence supporting mediation [40] and others showing support for only certain types of individuals. For example, Riggs et al [49] found that a web-based PFI, which included marijuana PBS, reduced marijuana use among a sample of college students who used it heavily. However, PBS use increased only among women who received PFI. The study described here has great potential to increase the efficacy of an important component of PFIs, as PBS skill training is one of the few components that reflect strategies for reducing use or consequences. Prince et al [36] examined an app used to collect event-level reports on marijuana use and PBS and found that event-level reductions in marijuana use were associated with greater PBS use. As this study used a single PBS item to assess the occurrence of any PBS use, it was not able to determine whether certain types of PBS were associated with less use or consequences. Thus, an essential step in determining the usefulness of alcohol and marijuana PBS on alcohol, marijuana, and CAM or SAM use is to conduct a rigorous pilot study of a PBS-focused intervention.

## Methods

### Study Design

This study will use an iterative process of focus groups and cognitive interviews (phase 1) to develop a novel web-based SMS text messaging intervention to be evaluated in a pilot randomized clinical trial (phase 2) to evaluate feasibility, acceptability, and preliminary effect sizes. In both phases, young adults from Texas, United States, aged 18 to 24 years, who typically use alcohol and marijuana at least twice per week, will be recruited.

### Ethics Approval

This study was reviewed and approved by the North Texas Regional Institutional Review Board (1679036-1). All study procedures were approved by the single institutional review board of record. All participants will sign an approved consent form in accordance with the ethical standards of Helsinki.

### Focus Groups and Cognitive Interviews (Phase 1)

Phase 1 will focus on examining motivations for alcohol and marijuana PBS use (and nonuse of PBS), as well as the quality of PBS use among young adults (aged 18-24 years) who use both alcohol and marijuana. We will conduct web-based focus groups (10 groups; N=10 per group) and cognitive interviews (N=10) to determine why young adults use or do not use specific PBS related to alcohol and marijuana use. Focus groups and cognitive interviews will discuss the level of the quality in which PBS are used and the various contexts in which PBS may or may not be used. All discussions will consider the use of either of the substances alone on a given day, as well as SAM or CAM use on a given day, and will address ways in which the motivations and quality of PBS could be incorporated into a web-based and SMS text messaging PBS intervention, as well as elicit feedback on drafted intervention material. The results of phase 1 will inform the development and delivery of the intervention to be tested in a pilot study (phase 2).

### Pilot Study (Phase 2)

#### Overview

In phase 2, we will conduct a pilot study—informed from phase 1 findings—of web-based, and SMS text messaging intervention with young adults (N=200; aged 18-24 years), who typically use alcohol and marijuana for at least 2 days per week to determine the feasibility, acceptability, and preliminary effect sizes. Participants will be randomized to either the intervention or waitlist control group*.* All participants will complete screening, baseline and daily morning surveys, and a 2-month follow-up. Participants in the intervention condition will receive a brief, web-based intervention focusing on self-selected alcohol and marijuana PBS messages and motives for using alcohol- and marijuana-related PBS. The intervention content will also be delivered via SMS text messages 3 days a week (random day, Friday, and Saturday) for 8 consecutive weeks. Participants in both conditions will report on PBS use, motivations for PBS use (and nonuse), quality of PBS use, and alcohol and marijuana use in morning surveys timed to occur the day after the intervention SMS text messages for those in the intervention group (morning after random day, Saturday, and Sunday).

The hypotheses for the phase 2 pilot randomized controlled trial are detailed in the following sections.

#### Hypothesis 1

We hypothesize that the intervention will be feasible (achieving the recruitment goal and doing so within an acceptable time frame; high study retention) and acceptable (enrolling a high proportion of eligible participants; obtaining favorable participants’ ratings of intervention components and ratings of accessibility, usability, convenience, and relevance, as measured after the web-based intervention and after SMS text message assessments).

#### Hypothesis 2

We expect that receiving the intervention will be associated with short-term (2-month) increases in PBS use, motivations for PBS use, and quality of PBS use, as well as decreases in motivations for PBS nonuse and reductions in past 2-month alcohol use, CAM and SAM use, and related consequences.

#### Hypothesis 3

Using event-level data, we expect that on days when individuals’ motivations to use PBS are elevated (ie, higher than their average level) or the quality of their PBS use is elevated (ie, higher than their average level), they will report lower alcohol use, be less likely to report CAM or SAM use, and report fewer negative consequences. These effects will be stronger in the intervention group than in the waitlist control group.

#### Hypothesis 4

Using event-level data, we will examine whether days when young adults use alcohol alone, compared with both CAM and SAM use days, are associated with greater use of alcohol PBS, greater motivation to use alcohol PBS, and higher quality of alcohol PBS use. Similarly, we will examine event-level associations between PBS use and consequences (alcohol and marijuana use) to determine whether PBS are as effective at reducing consequences when CAM or SAM use occurs.

### General Recruitment

For both phases, we will use a multimethod approach to reach a wide cross-section of young adults (aged 18-24 years) living in Texas, United States, who use alcohol and marijuana, such as in-person recruitment, flyers in businesses and community centers, web-based and newspaper advertisements, bus advertisements, and social media. Social media outreach will comprise a web-based Facebook fan page, which will provide a brief description of the study and links to the study website. We will use a combination of paid Facebook, Twitter, and Instagram advertisements and promote boosts on our study page to increase our web-based presence. Social media will be our main recruitment strategy; however, other strategies such as newspaper advertisements, Craigslist advertisements, Google advertisements, and flyers will be used to ensure that we reach participants who may not participate in social networking sites. Moreover, we will collaborate with local community organizations that have community outreach, such as local Young Men’s Christian Associations and substance use prevention organizations. All advertisements will provide a website address (URL) for more information and eligibility screening.

The initial screening will be conducted on the web to determine eligibility (Textbox 1). Eligibility questions will be embedded in demographic and behavioral questions to avoid making the criteria obvious. We aim to recruit an equal number of young adults at each age (eg, 18, 19, and 20) and sample based on the demographics of Texas. Our prior studies have successfully used similar procedures to recruit demographically diverse samples [55,56]. We have developed and programmed a system database for recruitment and tracking in our studies, which we will adapt for this research.

**Eligibility criteria for phase 1 and phase 2.**

**Inclusion criteria**

- Aged 18 to 24 years
- Live in Texas, United States
- Have a valid email address
- Own a cell phone number with SMS text messaging capabilities
- Okay with receiving SMS text messages
- Typically drink at least 2 days a week
- Typically use marijuana at least 2 days a week
- Report having at least one alcohol-related and one marijuana-related consequence in the past month
- Report being in contemplation or action stage based on readiness to change scale for alcohol or marijuana (ie, not in precontemplation stage)
- If female, not pregnant or trying to become pregnant
- Not currently in treatment for alcohol or substance use
- Willing to participate in a web-based focus group or cognitive interview via Zoom videoconferencing (criterion for phase 1 only)
- Device must meet the system requirements to participate in the web-based focus group or cognitive interview (eg, have iOS 8.0 or later and Android 4.0x or later or have another video-enabled device; criterion for phase 1 only)
- Willing to participate in a pilot study with daily morning surveys (criterion for phase 2 only)

### Phase 1: Focus Groups and Cognitive Interview Procedures

#### Overview

An iterative process of focus groups and cognitive interviews will inform the development and delivery of the intervention to be tested in the pilot study (aim 2). Focus groups and cognitive interviews will allow us to better elicit personally relevant reasons to make a change in one’s behavior and decide to engage in PBS for alcohol use or marijuana use so that we can optimize our SMS text messaging intervention. Results from the phase 1 focus groups will inform phase 2 by providing guidance on the specific motives behind PBS use or nonuse most commonly reported, informing the definitions and descriptions of the quality of PBS use to be presented to participants in phase 2, and making any additional changes to the proposed intervention materials or delivery based on participant feedback. The primary goal of the focus groups and cognitive interviews is to obtain participant feedback to help us develop an intervention that is clear, understandable, relevant, usable, and acceptable to the target population.

#### Focus Groups and Cognitive Interviews

A total of 10 focus groups will be run with approximately 10 people in each group, for a total of up to 100 individuals. For individuals who self-report as gender nonbinary, we will present two options from which the participant can select one: (1) their preferred gender focus group or (2) an individual cognitive interview. All focus groups and cognitive interviews will have a phenomenological focus, allowing for a free-flowing discussion among participants, with the flexibility to probe participants’ responses by the moderator. Focus groups and cognitive interviews will consider the use of alcohol or marijuana alone on a given day; CAM and SAM use on a given day; and how CAM and SAM use may affect motivation to use PBS, the type of PBS used (alcohol or marijuana), and the quality of PBS use. Focus groups and cognitive interviews will focus on the *why* behind using PBS as participants will be asked to discuss various ways in which the motivations for and quality of PBS use could be incorporated into a web-based and SMS text messaging PBS intervention, such as eliciting feedback on the use of drafted intervention material. Participants will be asked to provide reasons why they may choose not to use a certain PBS and what might motivate them to consider using the same strategy. Participants will be asked to discuss why some PBS are used more or less often and why they are used with lower or higher quality. These questions will provide the necessary information related to the motivations and reasons for using PBS, which will ultimately be targeted in the subsequent pilot study. The moderator will explore experiences of and reactions to the drafted intervention materials and will probe to see whether young adults, in general, would want to learn about opportunities to participate in an intervention study and gauge the interest for involvement in an intervention outside of a paid research study. Focus groups and cognitive interviews (60 minutes) will be conducted on the internet via Zoom videoconferencing software, and participants will receive US $50 compensation. Focus groups and cognitive interviews will be audiotaped using the Zoom recording feature. Audiotapes will also be transcribed using transcription software and checked by the team for any errors. Focus groups and cognitive interviews will be conducted in an iterative process, whereby, after the initial sessions, investigators will meet to decide what changes, if any, should be made before conducting the remaining focus groups and cognitive interviews.

### Phase 2: Pilot Randomized Controlled Trial Procedures

**Figure 1:**
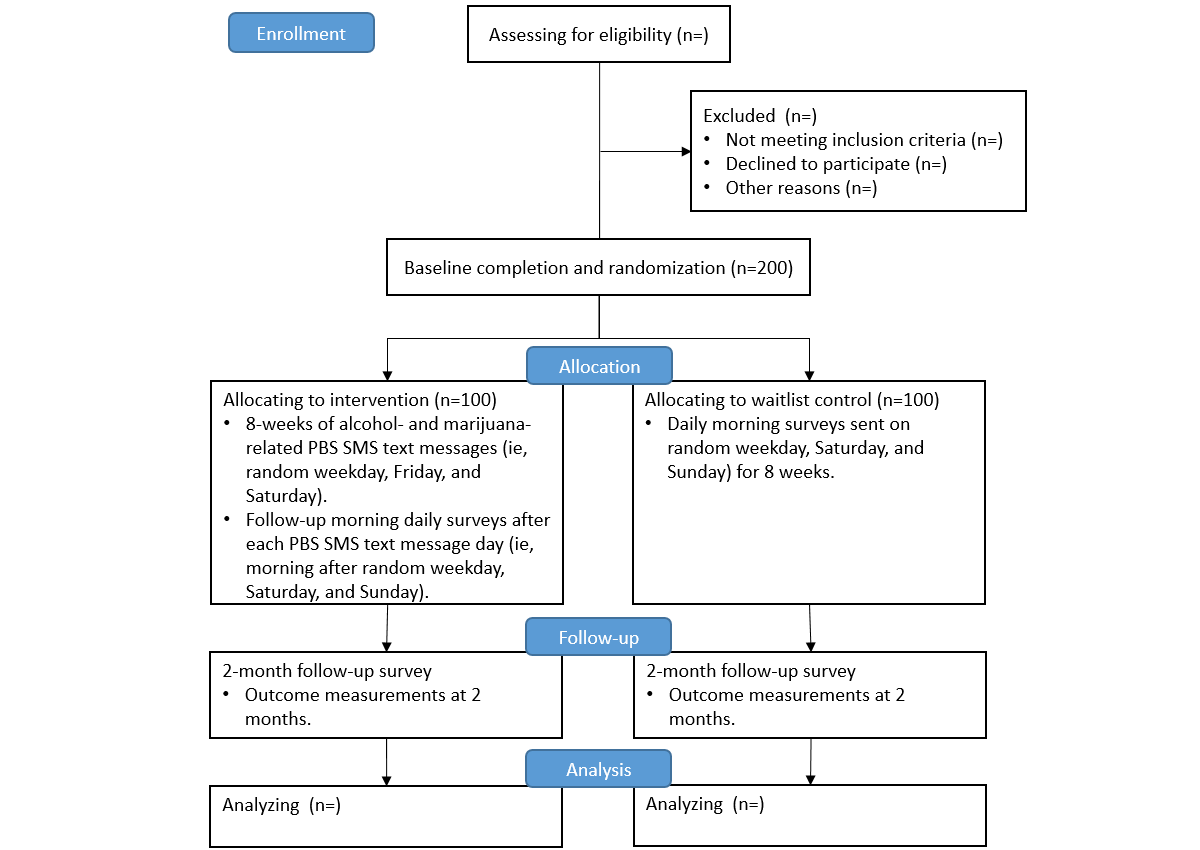
Randomized controlled trial workflow diagram. PBS: protective behavioral strategies.

Phase 2 will involve a pilot study with 200 young adults (aged 18-24 years) from Texas, United States, who typically drink alcohol and use marijuana for at least 2 days per week to determine the feasibility, acceptability, and preliminary effect sizes (Figure 1). Participants will be randomized to the web-based and SMS text messaging intervention (N=100) or waitlist control (N=100) groups. All participants will complete a screening survey, baseline assessment, a 2-month follow-up, as well 24 daily morning surveys over 8 weeks. All surveys will be administered on the web. Informed by the content and process details generated through focus groups and cognitive interviews in aim 1, participants in the intervention condition will receive a brief, web-based intervention that is self-administered and focuses on self-selected alcohol and marijuana PBS messages [57] and motives for using alcohol- and marijuana-related PBS. Participants will be prompted to choose 12 alcohol PBS and 12 marijuana PBS that they are motivated to use (from a list of possible PBS for alcohol and marijuana) and will identify whether they prefer to receive the PBS content during the week (random weekday) and on the weekend. For example, a participant could indicate that they are less likely to drink on a random weekday but more likely to use marijuana and, thus, would like the marijuana content during the week and the alcohol content on the weekend. Alternatively, a participant could indicate that they are likely to co-use alcohol and marijuana on weekends and, thus, would receive both types of PBS each weekend day. For each self-selected PBS, the web-based intervention will prompt them to provide information about why they selected that particular PBS.

The web-based intervention will also include examples related to how to use PBS in a high-quality manner to ensure that not only are PBS being used but that when they are used, the PBS are being used in an effective manner to affect substance use outcomes. For example, for the alcohol PBS specifying to *determine not to exceed a set number of drinks*, participants will navigate web-based content related to the process of deciding what is a safe limit to set based on personalized estimated blood alcohol concentration calculations. For the PBS to *use a designated driver,* the intervention content will emphasize the importance of ensuring that the designated driver has not had anything to drink or used any marijuana and that even one drink is too much. When participants select the marijuana PBS to *use a little and then wait to see how you feel before using more*, participants will be guided through deciding how much a *little* is and how long they should wait to see which effects they experience before using more marijuana. When they select *take periodic breaks if it feels like you are using marijuana too frequently,* they will be prompted by the web-based intervention to decide how they would know if they are using it too frequently and what kind of break would be ideal to actually reduce harm (eg, a few days, weeks, or months). The expected length of time to complete the self-administered web-based intervention is 20 minutes.

After completion of the web-based intervention, participants assigned to the intervention condition will receive a brief postintervention survey to assess the acceptability of the intervention content they received. Next, the intervention content will be delivered via SMS text messages 3 days a week (random day, Friday, and Saturday) over 8 consecutive weeks. On the days that participants will be receiving SMS text messaging intervention content, their day will start with 4 SMS text messages requesting a numerical reply consistent with other protocols assessing readiness and goal setting [58]. The first series of 2 SMS text messages will ask them to complete a readiness ruler assessing the importance of making a change in their alcohol use and, separately, marijuana use. Although the stem with the ruler during screening is *in general*, for these day-of-intervention SMS text messages, it will be *at this moment* (eg, “at this moment, on a scale of 0 [not important] to 10 [extremely important], how important is it for you to change your current drinking if you decided to?”). Then, the second series of 2 SMS text messages request a response to a modified readiness ruler assessing thoughts about openness to changing their behavior separately for alcohol and for marijuana (eg, “at this moment, on a scale of 0 [not at all] to 10 [extremely willing], how willing are you to try a new strategy around your alcohol use today?”). Later that day, the PBS content they receive will be personalized and matched to their importance or willingness ratings, such that SMS text messaging content is stage appropriate. For participants who rate a 0 to 3, typically precontemplation, they will receive a statement with a serious harm reduction PBS suggestion aiming to prompt contemplation of change with no action stage suggestion that they could or should do so (eg, “Eating before or during drinking slows absorption of alcohol & people find they can avoid unwanted consequences. Consider how this might work for you, if at all.”). Participants who rate a 4 to 6, which reflects ambivalence and contemplation, will receive an SMS text message with a small step, manner of drinking PBS suggestion (eg, “Drinking slowly can keep degree of intoxication from sneaking up on you. What might you be willing to try to slow down rate (if anything)? (a) pacing sips, (b) alternating sips/drinks with water, (c) something else, (d) I’m not sure this would work for me.”). Finally, participants who rate 7 to 10 will receive an action stage PBS reflecting stopping or limiting strategies (eg, “Determining not to exceed a set number of standard drinks can reduce unwanted effects. You said you’re ready to try something new and make a change in your drinking. What limit do you want to set for yourself tonight? Please text back the number of drinks you have in mind.”).

Participants in both conditions will report on PBS use and nonuse, including motivations for and quality of PBS use and alcohol and marijuana use in a morning survey that is timed to occur the day after the intervention messages (the morning after random day, Saturday, and Sunday). The waitlist control condition will not receive any intervention content during the 8-week period of data collection to support testing of the primary aims but will complete baseline, 2-month, and daily surveys according to the same schedule as the intervention group to assess event-level PBS use, PBS nonuse, alcohol and marijuana use, CAM and SAM use*,* and related consequences for up to 24 days over 8 weeks. All waitlist control participants will receive the intervention and postintervention survey at the end of the 2-month survey*.*

Participants will be compensated US $25 for the baseline survey, US $10 for postintervention assessment, up to US $58 for completion of all 24 daily surveys (US $2 per completed daily survey, with an additional US $10 bonus incentive for completing at least 90% of the surveys), and US $25 for a 2-month follow-up survey, for a total of up to US $118 across the study period.

### Phase 2 Measures

#### Baseline and 2-Month Measures

##### Demographics

Demographics will include, but are not limited to, sex assigned at birth, gender, age, height, weight, and living situation.

##### Alcohol Measures

Lifetime, past year, and past month alcohol use measures will include items from the Monitoring the Future (MTF) study [59]. Drinking will be assessed using the Daily Drinking Questionnaire (Cronbach α=.73) [60] and the Alcohol Use Disorders Identification Test (Cronbach α=.85) [61]. Negative consequences will be assessed using the Young Adult Alcohol Consequences Questionnaire (Cronbach α=.79) [62]. Alcohol PBS will be assessed using the Protective Behavioral Strategies Survey-20 (Cronbach α=.63-.81) [63]. Motivations for alcohol PBS use and nonuse will be assessed (Cronbach α=.80) [43,64]. The Readiness to Change Questionnaire (Treatment Version Revised) will be used to assess the readiness to change drinking habits [65,66].

##### Marijuana and Other Substance Use Measures

Marijuana use will be measured using MTF items such as lifetime, past year, and past month [59]. The Daily Marijuana Questionnaire will be used to assess marijuana use based on the typical number of hours spent high per day (Cronbach α=.97) [67]. The Marijuana Consequences Questionnaire [68] will measure a broad range of negative marijuana consequences (Cronbach α=.89). We will also administer the Marijuana Problem Scale (Cronbach α=.85) [69]. To assess risk for substance use disorder, we will use the Cannabis Use Disorders Identification Test-Revised (Cronbach α=.80) [70]. Marijuana PBS will be assessed using the PBS for Marijuana-36 (Cronbach α=.93) scale [34]. Other substance use will be assessed for lifetime and past month frequency using the Customary Drinking and Drug Use Record (Cronbach α=.70-.94) [71,72]. Motivations for marijuana PBS use and nonuse will be assessed using items parallel to the alcohol PBS motivation measure [43,64]. The Readiness to Change Questionnaire (Treatment Version Revised) will be adapted to assess readiness to change marijuana use [65,66].

Questions regarding SAM use will be adapted from MTF [73]: “On how many occasions (if any) during the last 30 days have you used alcohol and marijuana at the same time–that is, so that their effects overlapped?” CAM use will be determined from alcohol and marijuana measures (ie, endorsement of both alcohol and marijuana use within the same time frame) [67].

#### Acceptability Measures

A modified System Usability Scale [74-76] and Website Analysis and Measurement Inventory (WAMMI) [77] will assess the perceived usability of the web-based intervention and SMS text messages, perceived favorability of the web-based design, ease of navigation and use, convenience, relevance, and usefulness. The perceived engagement and appeal of the intervention and SMS text messages will also be assessed [78,79]. Participants will complete items to evaluate the web-based portion’s content (thought provoking, easy to understand, relevant, useful, motivation to change self or others, and open-ended questions on the most useful and engaging portion of the web-based feedback session) and format (attention grabbing, interesting, and enjoyable).

#### Daily Measures

Our strategy is to collect daily reports each morning after the intervention participants receive the SMS text messaging content. Each intervention participant will be yoked to a participant in the waitlist control group.

##### Yesterday’s Alcohol or Marijuana Use

Participants will report the number of standard drinks consumed on the previous day, the number of hours they spent drinking, whether they used marijuana, the number of sessions that they used marijuana, and how long they were high. SAM use will be assessed by asking, “Yesterday, did you use alcohol and marijuana at the same time–that is, so that their effects overlapped?” [14]. CAM use will be identified by the endorsement of alcohol and marijuana use the previous day but responding *no* to the SAM use item.

##### Substance-Related Consequences

The consequences experienced the previous day will be assessed using items from the alcohol and marijuana consequences scales. For alcohol, we will administer items used in our previous daily diary study on alcohol use [55]. A modified Marijuana Problem Scale [80] and Rutgers Marijuana Problem Index [81] will assess marijuana consequences, selecting acute items appropriate for daily-level measurements [82].

##### PBS Use and Quality

PBS use and quality on the previous day will be assessed by having participants report which, if any, alcohol and marijuana PBS they used the previous day and, for those they report using, how well they implemented the PBS (ie, quality) and how helpful they perceived the strategy to be.

##### Motivations to Use PBS

Motivations for each alcohol and marijuana PBS use will be assessed by asking open-ended questions on why they selected to use those strategies that day.

##### Readiness to Change

Participants’ readiness to change [66,83] will be assessed with “At this moment, on a scale of 0 to 10, how important is it for you to change your current drinking/marijuana use if you decided to?”

##### Feasibility and Acceptability

We will assess feasibility and acceptability (ie, participant responses after reading SMS text messaging content and whether alcohol or marijuana was being used when participants read the SMS text messages). SMS text messages will comprise a 2-way dialog to assess whether the participants read the message. Adherence will be calculated as the percentage of SMS text messages that prompted participants’ response [57]. Participants will respond by indicating helpfulness, likeability, thought provoking, and clarity (eg, 1=*not at all* to 5=*very*). We will track message timing and content to determine factors that may affect intervention efficacy and alcohol or marijuana use. We will examine the response rates to intervention SMS text messages on days of alcohol and marijuana use.

### Statistical Analysis

Before inferential statistics, univariate and bivariate descriptive statistics will be used to examine the distributions and simple associations among the variables. Preliminary analyses will include the nature of missing data and the identification of extreme values. The baseline equivalence of PBS, alcohol, and marijuana measures and demographic representation across conditions in phase 2 will be examined. Feasibility and acceptability will be the primary outcomes of phase 2. Behavioral alcohol and marijuana outcomes (PBS use, PBS motivation, PBS quality, alcohol use, alcohol consequences, marijuana use, marijuana consequences, CAM use, and SAM use) will provide estimates of the base rates and variance in the outcomes.

The feasibility and acceptability (*hypothesis 1*) of the intervention will be tested in several ways. First, feasibility will be established by (1) achieving the recruitment goal (N=200); (2) achieving the recruitment goal within 6 months; and (3) the rate of study retention being ≥90%, including the proportion of young adults who complete the intervention, the proportion of daily surveys completed, and the 2-month follow-up retention. The acceptability of the intervention will be determined by (1) the proportion of eligible young adults enrolled (80% of eligible young adults agreeing to participate); (2) ratings of individual intervention components, including both web and SMS text messaging content (rating content as favorable overall); (3) ratings of accessibility (acceptable length of intervention and acceptable timing of intervention delivery), usability (ease of viewing and navigating web-based intervention and SMS text messages), convenience (mode of intervention delivery), and relevance of intervention content (engaging and helpful content); and (4) the proportion of young adults who would recommend the program (outside of a paid research study). Acceptability will be achieved if 80% of the responses in each domain are rated ≥4 (out of 5). For the System Usability Scale, scores <4.0 on the 5-point items indicate a need to re-examine intervention features, and scores of ≥68 on the 100-point total support overall usability. In the case that intervention areas do not meet these criteria, the investigative team will revise the intervention components before conducting a future large-scale randomized trial. The WAMMI comprises 20 validated statements used to evaluate websites and intervention programs. We will use this measure to assess the acceptability of our web-based and SMS text messaging intervention. Each statement is rated on a 5-point scale from strongly agree to disagree, and scores will be calculated for attractiveness, controllability, efficiency, helpfulness, and learnability, as well as the overall global usability score. All scores will be automatically calculated by the WAMMI website and compared with a large international database of scores for other projects. A global usability score of ≥50 indicates that a given website or intervention program is above average (50), according to a large international database maintained by the creators of the WAMMI.

For *hypothesis 2,* given the repeated measures design, generalized linear mixed models (GLMMs) [84,85] will be used. GLMMs (ie, hierarchical generalized linear models) allow for nonnormal outcomes (eg, count outcomes such as the number of days high or the number of negative consequences) and missing data, handle varying time points, and accommodate time-varying and time-invariant covariates. The models include two repeated measures (baseline and 2 months), yielding up to 400 observations (level 1: repeated measures) across 200 individuals (level 2: people; n=100 per condition). To test the intervention effects, the intervention condition will be a dummy variable that compares the intervention condition to the waitlist control condition (reference category). Of particular interest are the parameters that reflect the interaction between the intervention conditions and time*.* For count outcomes (eg, alcohol use and consequences), the outcome is connected to covariates through a log link function, which is the standard link function for Poisson GLMMs. Covariates can be exponentiated to yield rate ratios that describe the proportional change in the count outcome associated with a 1-unit increase in the covariate. If data show overdispersion when the variance exceeds the mean, the model will be extended to include a scale parameter to fit an overdispersed Poisson, or we will consider zero-altered models to ensure accurate inferences [86]. Sex assigned at birth, age, and baseline readiness to change will be included as covariates in all the analyses.

Both *hypothesis 3* and *hypothesis 4* use event-level data and can be tested with GLMMs, which are also used for *hypothesis 2*. The 2-level model accounts for the clustering of observations, whereby morning surveys (level 1: day-level) are nested within individuals (level 2: person-level). GLMMs can accommodate unequal observations per person. We will use an appropriate modeling distribution for all outcomes (eg, a zero-inflated Poisson distribution for count outcomes such as consequences and normal distribution for PBS motivation). We will evaluate whether the model assumptions are met (eg, normality of error terms) so that the data are modeled appropriately [86]. Centering of predictors and controlling for the associated higher-level effects will be performed based on standard practice and current recommendations. Sex assigned at birth and age will be person-level covariates in all analyses, and daily-level covariates will be alcohol use, marijuana use, weekends, and readiness to change in all analyses. Owing to the large number of models, *P* values will be adjusted [87].

Event-level designs using daily surveys produce rich and complex data sets that permit the examination of different types of associations among constructs, and these complex associations can be tested using GLMMs. For instance, *hypothesis 3* specifies that on days when individuals’ motivations to use PBS are elevated (ie, higher than their average level), they will report lower alcohol use. Here, PBS motivation is the predictor (person-centered), and the number of drinks consumed that day is the outcome. A cross-level interaction between the predictor (level 1) and condition (level 2) can be tested to determine whether this effect is stronger among those in the intervention condition than in the waitlist control condition. GLMM specifications can easily be modified for event-level data to test all the hypotheses specified by *hypothesis 3* and *hypothesis 4*. For instance, in *hypothesis 4*, each day will be coded as neither alcohol nor marijuana, alcohol alone, marijuana alone, CAM, or SAM. Then, dummy codes will be created to make specific comparisons (eg, alcohol alone days vs SAM use days).

## Results

This research was funded in May 2021 and approved by institutional review board in March 2021. Recruitment and enrollment for phase 1 began in January 2022. The findings of phase 1 will inform the development of novel web-based and SMS text messaging interventions that will be tested in phase 2. Phase 2 is anticipated to begin in January 2023. The findings will be published in peer-reviewed journals and presented at international, national, or regional professional meetings and conferences.

## Discussion

### Principal Findings

The most successful young adult alcohol or marijuana interventions involve the provision of accurate, nonjudgmental, and personalized feedback [88]; however, notably, the inclusion and effectiveness of PBS content are inconsistent [54]. Moreover, the active components of brief interventions are not well understood [89], and findings have been inconclusive regarding whether PBS mediates the intervention efficacy of college student PFIs, with only some studies showing evidence of mediation [40]. A possible reason for these findings is that we often do not know young adults’ motivations for using (or not using) PBS or the quality of PBS use across individuals or across drinking occasions. This study will provide an in-depth examination of which PBS young adults are motivated to use (including implementation quality) and the reasons that young adults may or may not use PBS. Understanding why young adults choose not to use PBS on specific occasions or do not engage in effective or high-quality PBS use on certain occasions has significant clinical implications, whereby interventions may need to spend more time increasing motivations to use PBS in an effective manner or work on reducing the perceived barriers (ie, reasons individuals are not using PBS). Clinicians may then be better able to work with young adults in various settings (eg, campus counseling and health centers, residence halls, health and wellness services, and community mental health clinics) to reduce or prevent excessive alcohol and marijuana use and related negative consequences. This study has great potential for making a substantial impact in the field and public health (particularly as more states permit legal access to marijuana for those aged ≥21 years) as it will address the problem of high importance (young adult alcohol and marijuana use) by being the first to develop and refine a PBS intervention that specifically focuses on the motivations for alcohol and marijuana PBS use and nonuse, as well as the quality of use, which is an overlooked aspect of current PBS-related intervention approaches.

### Limitations

Although this study will use a strict application of the scientific method to achieve robust, unbiased, and replicable results via several design features, including explicit inclusion and exclusion criteria, study design (randomization and inclusion of a waitlist control), and data analytic plans, there are several potential limitations that need to be acknowledged. First, the use of incentives in research may lead to selection effects that could have an impact on external validity. A meta-analysis found small effects of incentives to increase recruitment for web-based research [90]. However, selection effects are typically not a problem for randomized trials, as random assignment ensures relatively similar characteristics across study conditions [91]. However, we will include questions in the focus groups to assess what would make participants willing to participate in a similar study without monetary incentives. A second potential limitation of this research is that we will collect data in a single state that does not currently have legalized marijuana. Thus, we will not be able to directly test how legalization influences alcohol and marijuana use and related PBS in young adult populations. Furthermore, as this is a small-scale pilot study, it was not designed to be fully powered; however, the results of this study will provide preliminary effect sizes to calculate power for a subsequent full-scale randomized controlled trial.

### Conclusions

This study will fill critical gaps in the literature by identifying the extent to which motivations for PBS use and nonuse (marijuana or alcohol) and the quality of PBS use (degree of effectiveness or degree of implementation) differ when using alcohol alone versus concurrently or simultaneously with marijuana. The overall goal of this study is to inform a pilot study of a newly developed alcohol and marijuana PBS intervention. This research will (1) collect pilot data to establish the feasibility and acceptability and test the web-based and SMS text messaging PBS intervention (baseline and 2 months) and (2) collect event-level data to examine daily-level associations among PBS motivation and quality, PBS use and nonuse, alcohol and marijuana use, and negative consequences, with a focus on how PBS may differ on CAM or SAM use days compared with alcohol-only days. This study will provide an in-depth understanding of young adults’ PBS use and has the potential to develop a more efficacious intervention for these co-occurring or SAM behaviors.

## Appendix

Multimedia Appendix 1 Peer-review report form the Epidemiology, Prevention and Behavior Research Review Subcommittee, National Institute on Alcohol Abuse and Alcoholism Initial Review Group (National Institutes of Health, USA).

## Abbreviations

CAM: concurrent alcohol and marijuana
GLMM: generalized linear mixed model
MTF: Monitoring the Future
NIAAA: National Institute on Alcohol Abuse and Alcoholism
PBS: protective behavioral strategies
PFI: personalized feedback intervention
SAM: simultaneous alcohol and marijuana
WAMMI: Website Analysis and Measurement Inventory
