# Supplementary material for: Investigating Why and How Young Adults Use Protective Behavioral Strategies for Alcohol and Marijuana Use: Protocol for Developing a Randomized Controlled Trial
Source: JMIR Res Protoc. 2022 Apr 19;11(4):e37106. doi: 10.2196/37106 (PMC9066324; doi:10.2196/37106)
Supplement: Multimedia Appendix 1 [file resprot_v11i4e37106_app1.pdf]

Notice of Award

FAIN# R34AA028730
Federal Award Date
04/21/2021

Recipient Information

1. Recipient Name
UNIVERSITY OF NORTH TEXAS HEALTH SCIENCE CENTER AT FORT WORTH
3500 CAMP BOWIE BLVD

FORT WORTH, TX 76107

2. Congressional District of Recipient
12

3. Payment System Identifier (ID)
1756064033A1

4. Employer Identification Number (EIN)
756064033

5. Data Universal Numbering System (DUNS)
110091808

6. Recipient's Unique Entity Identifier

7. Project Director or Principal Investigator
Melissa A Lewis, PHD
Professor
melissa.lewis@unthsc.edu
817-735-5136

8. Authorized Official
Angelita Trevino
ospext@unthsc.edu
817-735-5073

Federal Agency Information

9. Awarding Agency Contact Information
Lauren Early
Grants Management Specialist
NATIONAL INSTITUTE ON ALCOHOL ABUSE AND ALCOHOLISM
earlyle@mail.nih.gov
301-443-2434

10. Program Official Contact Information
MICHAEL E. HILTON
Program Director
NATIONAL INSTITUTE ON ALCOHOL ABUSE AND ALCOHOLISM
mhilton@mail.nih.gov
301-402-9402

Federal Award Information

11. Award Number
1R34AA028730-01A1

12. Unique Federal Award Identification Number (FAIN)
R34AA028730

13. Statutory Authority
42 USC 241 42 CFR 52

14. Federal Award Project Title
Examining Motivations for and Quality of Alcohol and Marijuana Protective Behavior Strategy Use: Improving Prevention of Hazardous Young Adult Substance Use

15. Assistance Listing Number
93.273

16. Assistance Listing Program Title
Alcohol Research Programs

17. Award Action Type
New Competing

18. Is the Award R&D?
Yes

Summary Federal Award Financial Information

**19. Budget Period Start Date** 05/01/2021 **– End Date** 03/31/2022	
**20. Total Amount of Federal Funds Obligated by this Action**	$204,034
20 a. Direct Cost Amount	$140,001
20 b. Indirect Cost Amount	$64,033
**21.** Authorized Carryover	$0
**22.** Offset	$0
**23.** Total Amount of Federal Funds Obligated this budget period	$204,034
**24. Total Approved Cost Sharing or Matching, where applicable**	$0
**25. Total Federal and Non-Federal Approved this Budget Period**	$204,034
**26. Project Period Start Date** 05/01/2021 **– End Date** 03/31/2024	
**27.** Total Amount of the Federal Award including Approved Cost Sharing or Matching this Project Period	$204,034

28. Authorized Treatment of Program Income
Additional Costs

29. Grants Management Officer - Signature
Judy Fox

30. Remarks
Acceptance of this award, including the "Terms and Conditions," is acknowledged by the recipient when funds are drawn down or otherwise requested from the grant payment system.

CLINICAL TRIAL PLANNING GRANT
Department of Health and Human Services
National Institutes of Health

NATIONAL INSTITUTE ON ALCOHOL ABUSE AND ALCOHOLISM

SECTION I – AWARD DATA – 1R34AA028730-01A1

Principal Investigator(s):
Melissa A Lewis, PHD

Award e-mailed to: ospext@unthsc.edu

Dear Authorized Official:

The National Institutes of Health hereby awards a grant in the amount of $204,034 (see "Award Calculation" in Section I and "Terms and Conditions" in Section III) to UNIVERSITY OF NORTH TEXAS HLTH SCI CTR in support of the above referenced project. This award is pursuant to the authority of 42 USC 241 42 CFR 52 and is subject to the requirements of this statute and regulation and of other referenced, incorporated or attached terms and conditions.

Acceptance of this award, including the "Terms and Conditions," is acknowledged by the recipient when funds are drawn down or otherwise requested from the grant payment system.

Each publication, press release, or other document about research supported by an NIH award must include an acknowledgment of NIH award support and a disclaimer such as "Research reported in this publication was supported by the National Institute On Alcohol Abuse And Alcoholism of the National Institutes of Health under Award Number R34AA028730. The content is solely the responsibility of the authors and does not necessarily represent the official views of the National Institutes of Health." Prior to issuing a press release concerning the outcome of this research, please notify the NIH awarding IC in advance to allow for coordination.

Award recipients must promote objectivity in research by establishing standards that provide a reasonable expectation that the design, conduct and reporting of research funded under NIH awards will be free from bias resulting from an Investigator's Financial Conflict of Interest (FCOI), in accordance with the 2011 revised regulation at 42 CFR Part 50 Subpart F. The Institution shall submit all FCOI reports to the NIH through the eRA Commons FCOI Module. The regulation does not apply to Phase I Small Business Innovative Research (SBIR) and Small Business Technology Transfer (STTR) awards. Consult the NIH website http://grants.nih.gov/grants/policy/coi/ for a link to the regulation and additional important information.

If you have any questions about this award, please direct questions to the Federal Agency contacts.

Sincerely yours,

Judy Fox
Grants Management Officer
NATIONAL INSTITUTE ON ALCOHOL ABUSE AND ALCOHOLISM

Additional information follows

Cumulative Award Calculations for this Budget Period (U.S. Dollars)

**Federal Direct Costs**	$140,001
**Federal F&A Costs**	$64,033
**Approved Budget**	$204,034
**Total Amount of Federal Funds Authorized (Federal Share)**	$204,034
**TOTAL FEDERAL AWARD AMOUNT**	$204,034
**AMOUNT OF THIS ACTION (FEDERAL SHARE)**	$204,034

**SUMMARY TOTALS FOR ALL YEARS** (for this Document Number)		
**YR**	**THIS AWARD**	**CUMULATIVE TOTALS**
1	$204,034	$204,034
2	$195,979	$195,979
3	$193,987	$193,987

Recommended future year total cost support, subject to the availability of funds and satisfactory progress of the project

Fiscal Information:

**Payment System Identifier:**	1756064033A1
**Document Number:**	RAA028730A
**PMS Account Type:**	P (Subaccount)
**Fiscal Year:**	2021

IC	CAN	2021	2022	2023
AA	8484370	$204,034	$195,979	$193,987

Recommended future year total cost support, subject to the availability of funds and satisfactory progress of the project

NIH Administrative Data:
PCC: AP C / **OC**: 41021 / **Released**: Fox, Judy 04/14/2021
Award Processed: 04/21/2021 12:14:55 AM

SECTION II – PAYMENT/HOTLINE INFORMATION – 1R34AA028730-01A1

For payment and HHS Office of Inspector General Hotline information, see the NIH Home Page at http://grants.nih.gov/grants/policy/awardconditions.htm

SECTION III – STANDARD TERMS AND CONDITIONS – 1R34AA028730-01A1

This award is based on the application submitted to, and as approved by, NIH on the above-titled project and is subject to the terms and conditions incorporated either directly or by reference in the following:

a. The grant program legislation and program regulation cited in this Notice of Award.
b. Conditions on activities and expenditure of funds in other statutory requirements, such as those included in appropriations acts.
c. 45 CFR Part 75.
d. National Policy Requirements and all other requirements described in the NIH Grants Policy Statement, including addenda in effect as of the beginning date of the budget period.
e. Federal Award Performance Goals: As required by the periodic report in the RPPR or in the final progress report when applicable.
f. This award notice, INCLUDING THE TERMS AND CONDITIONS CITED BELOW.

(See NIH Home Page at http://grants.nih.gov/grants/policy/awardconditions.htm for certain references cited above.)

Research and Development (R&D): All awards issued by the National Institutes of Health (NIH) meet the

definition of "Research and Development" at 45 CFR Part§ 75.2. As such, auditees should identify NIH awards as part of the R&D cluster on the Schedule of Expenditures of Federal Awards (SEFA). The auditor should test NIH awards for compliance as instructed in Part V, Clusters of Programs. NIH recognizes that some awards may have another classification for purposes of indirect costs. The auditor is not required to report the disconnect (i.e., the award is classified as R&D for Federal Audit Requirement purposes but non-research for indirect cost rate purposes), unless the auditee is charging indirect costs at a rate other than the rate(s) specified in the award document(s).

An unobligated balance may be carried over into the next budget period without Grants Management Officer prior approval.

This grant is subject to Streamlined Noncompeting Award Procedures (SNAP).

This award is subject to the requirements of 2 CFR Part 25 for institutions to receive a Dun & Bradstreet Universal Numbering System (DUNS) number and maintain an active registration in the System for Award Management (SAM). Should a consortium/subaward be issued under this award, a DUNS requirement must be included. See http://grants.nih.gov/grants/policy/awardconditions.htm for the full NIH award term implementing this requirement and other additional information.

This award has been assigned the Federal Award Identification Number (FAIN) R34AA028730. Recipients must document the assigned FAIN on each consortium/subaward issued under this award.

Based on the project period start date of this project, this award is likely subject to the Transparency Act subaward and executive compensation reporting requirement of 2 CFR Part 170. There are conditions that may exclude this award; see http://grants.nih.gov/grants/policy/awardconditions.htm for additional award applicability information.

In accordance with P.L. 110-161, compliance with the NIH Public Access Policy is now mandatory. For more information, see NOT-OD-08-033 and the Public Access website: http://publicaccess.nih.gov/.

This award provides support for one or more clinical trials. By law (Title VIII, Section 801 of Public Law 110-85), the "responsible party" must register "applicable clinical trials" on the ClinicalTrials.gov Protocol Registration System Information Website. NIH encourages registration of all trials whether required under the law or not. For more information, see http://grants.nih.gov/ClinicalTrials_fdaaa/

In accordance with the regulatory requirements provided at 45 CFR 75.113 and Appendix XII to 45 CFR Part 75, recipients that have currently active Federal grants, cooperative agreements, and procurement contracts with cumulative total value greater than $10,000,000 must report and maintain information in the System for Award Management (SAM) about civil, criminal, and administrative proceedings in connection with the award or performance of a Federal award that reached final disposition within the most recent five-year period. The recipient must also make semiannual disclosures regarding such proceedings. Proceedings information will be made publicly available in the designated integrity and performance system (currently the Federal Awardee Performance and Integrity Information System (FAPIIS)). Full reporting requirements and procedures are found in Appendix XII to 45 CFR Part 75. This term does not apply to NIH fellowships.

Treatment of Program Income:

Additional Costs

SECTION IV – AA SPECIFIC AWARD CONDITIONS – 1R34AA028730-01A1

Clinical Trial Indicator: Yes

This award supports one or more NIH-defined Clinical Trials. See the NIH Grants Policy Statement Section 1.2 for NIH definition of Clinical Trial.

Based on a review of your application and the need to effect NIAAA budgetary and programmatic goals, your requested direct cost funding has been adjusted.

SALARY LIMITATION: None of the funds in this award shall be used to pay the salary of an individual at a rate in excess of the applicable salary cap. Therefore, this award and/or future years are adjusted accordingly, if applicable. Current salary cap levels can be found at http://grants1.nih.gov/grants/policy/salcap_summary.htm.

MODULAR AWARD: This is a Modular Grant Award without direct cost categorical breakdowns issued in accordance with the guidelines published in the current NIH Grants Policy Statement (see http://grants.nih.gov/grants/policy/policy.htm#gps).

CONSORTIA: This award includes funds awarded for consortium activity with **UNIVERSITY OF WASHINGTON**. Consortia are to be established and administered as described in the NIH Grants Policy Statement (NIH GPS).

DATA AND SAFETY MONITORING: This grant has been identified as requiring a Data andSafety Monitoring Plan (DSMP) in accordance with the NIAAA Data and Safety Monitoring Guidelines at http://www.niaaa.nih.gov/research/guidelines-and-resources/data-and-safety-monitoring-guidelines. The NIAAA Program Official named below has approved the DSMP as submitted by the grant recipient. Any changes in the DSMP must be reviewed and approved by the NIAAA Program Official.

DISSEMINATION PLAN: The clinical trial(s) supported by this award is subject to the plan submitted to NIH **07/31/2020** and the NIH policy on *Dissemination of NIH-Funded Clinical Trial Information.* The policy states that the clinical trial(s) funded by a NIH award will be registered in ClinicalTrials.gov not later than 21 calendar days after enrollment of the first participant and primary summary results reported in ClinicalTrials.gov, not later than one year after the completion date. The reporting of summary results is required by this term of award even if the primary completion date occurs after the period of performance.

This award is subject to additional certification requirements with each submission of the Annual, Interim, and Final Research Performance Progress Report (RPPR). The recipient must agree to the following annual certification when submitting each RPPR. By submitting the RPPR, the AOR signifies compliance, as follows:

In submitting this RPPR, the SO (or PD/PI with delegated authority), certifies to the best of his/her knowledge that, for all clinical trials funded under this NIH award, the recipient and all investigators conducting NIH-funded clinical trials are in compliance with the recipient's plan addressing compliance with the NIH Policy on Dissemination of NIH-Funded Clinical Trial Information. Any clinical trial funded in whole or in part under this award has been registered in ClinicalTrials.gov or will be registered not later than 21 calendar days after enrollment of the first participant. Summary results have been submitted to ClinicalTrials.gov or will be submitted not later than one year after the completion date, even if the completion date occurs after the period of performance.

NIAAA DATA ARCHIVE (NIAAADA) DATA SHARING PLAN: This award is subject to the data sharing guidance outlined in NOT-AA-19-02010 (https://grants.nih.gov/grants/guide/notice-files/NOT-AA-19-020.html). The Recipient agrees to adhere to the NIAAADA Data Sharing Plan (DSP) as approved by the NIAAA Program Officer assigned to this award. Dissemination of study data will be in accord with the Recipient's approved DSP. Please note that a statement of progress on the DSP must be included in the Research Performance Progress Report (RPPR; http://grants.nih.gov/grants/rppr/index.htm) under section C.5 "Other Products and Resource Sharing". Failure to adhere to the DSP as mutually agreed upon by the Recipient and the NIAAA may result in Enforcement Actions as described in the NIH Grants Policy Statement (https://grants.nih.gov/policy/nihgps/index.htm)

Complete NIAAADA Data Sharing Terms and Conditions can be found at https://nda.nih.gov/contribute_data_sharing_regimen.html.

INFORMATION: In order to redistribute awards more evenly throughout the year, budget periods are being adjusted. This award is issued with a **shortened initial budget period** and with 12 months of support. Continuation awards will cycle each year on **04/01.** The noncompeting continuation Research Performance Progress Report (RPPR) is due the 15th of the month preceding the month in which the budget period ends.

INFORMATION: The recycling of this award has changed the receipt date for the next competing continuation (type 2) application if applicable. Consult "application receipt, review and award schedule" in the NIH Grants Policy Statement (http://grants.nih.gov/grants/policy/policy.htm#gps) for the established deadline date.

SPREADSHEET SUMMARY
AWARD NUMBER: 1R34AA028730-01A1

INSTITUTION: UNIVERSITY OF NORTH TEXAS HLTH SCI CTR

Budget	Year 1	Year 2	Year 3
TOTAL FEDERAL DC	$140,001	$138,568	$140,306
TOTAL FEDERAL F&A	$64,033	$57,411	$53,681
TOTAL COST	$204,034	$195,979	$193,987

Facilities and Administrative Costs	Year 1	Year 2	Year 3
F&A Cost Rate 1	46%	48%	48%
F&A Cost Base 1	$56,566	$119,606	$111,836
F&A Costs 1	$26,020	$57,411	$53,681
F&A Cost Rate 2	48%		
F&A Cost Base 2	$79,193		
F&A Costs 2	$38,013		

SUMMARY STATEMENT
(Privileged Communication)

PROGRAM CONTACT:
MICHAEL HILTON
301-402-9402
mhilton@mail.nih.gov

Release Date: **11/18/2020**
Revised Date:

Application Number: **1 R34 AA028730-01A1**
Formerly: **1R34AA028730-01**

Principal Investigator

LEWIS, MELISSA A

Applicant Organization: UNIVERSITY OF NORTH TEXAS HLTH SCI CTR

Review Group: **AA-2**
Epidemiology, Prevention and Behavior Research Review Subcommittee

Meeting Date: **10/19/2020**
Council: **JAN 2021**
Requested Start: **04/01/2021**
RFA/PA: **PA18-775**
PCC: **AP C**

Project Title: **Examining Motivations for and Quality of Alcohol and Marijuana Protective Behavior Strategy Use: Improving Prevention of Hazardous Young Adult Substance Use**
SRG Action: **Impact Score:14**
Next Steps: **Visit https://grants.nih.gov/grants/next_steps.htm**
Human Subjects: **30-Human subjects involved - Certified, no SRG concerns**
Animal Subjects: **10-No live vertebrate animals involved for competing appl.**
Gender: **1A-Both genders, scientifically acceptable**
Minority: **1A-Minorities and non-minorities, scientifically acceptable**
Age: **7A-Only Adults, scientifically acceptable**

Project Year	Direct Costs Requested	Estimated Total Cost
1	150,000	219,998
2	150,000	219,998
3	150,000	219,998
TOTAL	450,000	659,993

ADMINISTRATIVE BUDGET NOTE: The budget shown is the requested budget and has not been adjusted to reflect any recommendations made by reviewers. If an award is planned, the costs will be calculated by Institute grants management staff based on the recommendations outlined below in the COMMITTEE BUDGET RECOMMENDATIONS section.

1R34AA028730-01A1 Lewis, Melissa

RESUME AND SUMMARY OF DISCUSSION: The PI of this resubmitted R34 application proposes to develop an online and text message-based protective behavioral strategies (PBS) intervention in Concurrent Alcohol and Marijuana (CAM) and/or Simultaneous Alcohol and Marijuana (SAM) young-adult users. The application has retained its previous strengths. These included significant research topic and exceptional team with successful track record of collaboration in developing and implementing interventions. The applicant was responsive to the previous concerns. The majority reviewers did not find additional drawbacks in the proposal. Some believed that individualized nature of motivations to use PBS is not fully taken into consideration by the investigators raising minor concerns with feasibility. The panel unanimously believed that this research, if carried out successfully, will lead to the development of efficacious interventions for SAM and/or CAM users. The proposal was rated as having a potential for a High Impact and was placed between Exceptional and Outstanding range.

DESCRIPTION (provided by applicant): Young adulthood is associated with increased alcohol and marijuana use compared to other developmental periods (Schulenberg et al., 2019). Alcohol and marijuana use place individuals at high risk for acute and long- term negative consequences (Volkow et al., 2014; White & Hingson, 2014). SAM use is associated with increased risk for consequences, compared to marijuana use alone or CAM use (Lipperman-Kreda et al., 2017). In the proposed research, all participants will be current alcohol and marijuana users allowing us to consider Concurrent Alcohol and Marijuana (CAM) use and Simultaneous Alcohol and Marijuana (SAM) use. In this application, CAM use refers to use of both substances on the same day, but not so their effects overlap, whereas SAM use refers to use of alcohol and marijuana at the same time so that their effects overlap. Despite the relatively large cross-sectional and longitudinal literature on protective behavioral strategies (PBS; Pearson, 2013), little is known about why young adults choose to use PBS on specific occasions or why young adults might use PBS differently across occasions. Moreover, findings have been mixed for PBS use as a mediator of intervention effects (Reid & Carey, 2015). Thus, there is significant room for improvement in the conceptualization, application, and understanding of alcohol and marijuana PBS. The proposed research is needed to determine motivations, or reasons, for when, why, and how young adults may or may not use PBS in a quality manner when using alcohol and/or marijuana. This gained knowledge can be used to enhance intervention efficacy by addressing these motivations in intervention content delivered across days. Research has yet to examine how alcohol and marijuana PBS use on a given day relates to an individual's use of alcohol or marijuana alone in comparison to CAM or SAM days. The current study has potential to contribute significantly to the literature as it will allow for a fine-grained examination of how alcohol and marijuana PBS use relate to alcohol, marijuana, and CAM/SAM use at the daily level. Taken together, the proposed study will fill these gaps by utilizing online focus groups and cognitive interviews to identify "why" young adults use or do not use PBS (for marijuana or alcohol) as well as to examine quality of PBS use and if quality differs on alcohol-only days, CAM days, or SAM days (Aim 1). Findings from Aim 1 will inform a pilot study (Aim 2) testing a newly developed online and text message alcohol and marijuana PBS intervention among alcohol and marijuana users age 18-24. The pilot study includes event-level data collection over 8 weekends to determine whether alcohol or marijuana PBS are as effective at reducing use or consequences when CAM or SAM use occurs, compared to using alcohol alone. In the pilot study (Stage I; N=200), we will establish acceptability, feasibility, and preliminary effect sizes to determine readiness for a full-scale efficacy trial. Because the proposed intervention will be designed to target PBS for alcohol and marijuana use the intervention will be relevant to a wider group of at-risk young adults.

PUBLIC HEALTH RELEVANCE

Young adult alcohol and marijuana use is a significant public health concern. This research will advance the field by contributing to our understanding of the role of protective behavioral strategy use

in the etiology and prevention of alcohol and marijuana use. The proposed application fits within NIAAA's and NIDA's strategic plans and has significant implications for public health due to the development and piloting of an intervention targeting alcohol and marijuana use, including concurrent and simultaneous use, for young adults by focusing on the optimal implementation of protective behavioral strategies across occasions.

CRITIQUE 1

Significance: 2
Investigator(s): 1
Innovation: 1
Approach: 2
Environment: 1

Overall Impact:

This resubmission proposes to develop and refine an online and text message-based protective behavioral strategies (PBS) intervention relevant for young adult alcohol and marijuana users. The application appears to be very responsive to the prior critiques and makes a strong case for the proposed research by addressing multiple gaps in the extant literature. The scientific premise is sound, the targeted constructs add to the literature, and the proposed methodology appears feasible. The investigative team has extensive experience with all major aspects of the proposed work. The potential public health impact in light of existing interventions is less clear but may be reasonable given the scientific rigor and developmental nature of the work.

1. Significance:

Strengths

- Young adult alcohol and marijuana use remain a significant public health concern, particularly when these two substances are used together.
- The dissemination of the intervention via online and text-based messaging should maximize its impact for a young adult population.
- The proposal clearly indicates how the research will address a number of gaps in the existing literature on interventions for young adult substance use.

Weaknesses

- It is not clear how much the proposed intervention development has the potential to impact young adult alcohol and marijuana use beyond the plethora of existing young adult interventions (many of which are being conducted by members of this research team). However, the innovation and scientific rigor of the proposal mitigate this concern considerably.

2. Investigator(s):

Strengths

- The PI and Co-Investigators all have extensive experience conducting substance use interventions in young adults, including online and text-based interventions. Generally, this is an exceptionally strong team for conducting the proposed research

Weaknesses

- It is not clear whether any of the investigators have experience with the nuanced characterizations associated with non-binary alcohol and marijuana use.

3. Innovation:

Strengths

- The in-depth focus on the use of and motivations for using protective behavioral strategies is innovative.
- Examining young adults use of PBS for marijuana use as well as concurrent and simultaneous alcohol and marijuana use at the daily level is also novel.
- The inclusion of non-binary gender is innovative although it is unclear how this will be characterized.

Weaknesses

- None noted.

4. Approach:

Strengths

- The pilot study design allows for more finely identifying and delineating key elements such as concurrent vs. simultaneous alcohol and marijuana use, motivations for the use of specific PBS, readiness for change, among others, which should provide a strong foundation for subsequent R01 scale investigation.
- Use of well-established indices for all major constructs.
- Event-level assessment is ideal for addressing the major foci that make the project innovative.
- Plans for assessing factors relevant for the development of intervention (e.g., adherence, feasibility, and acceptability) are detailed and well-organized.

Weaknesses

- The proposal might benefit from more detail on how non-binary gender will be characterized, whether recruitment will target such individuals, whether the inclusion of non-gender binary folks may have implications for the refinement of the assessment of CAM/SAM use and motivations to use PBS.
- The stringent inclusion criteria generally promote feasibility and scientific rigor, but may limit generalization (e.g., requiring a valid text messaging plan, stable internet access for focus groups or interviews, etc.)

5. Environment:

Strengths

- The STARR lab within the School of Public Health at the University of North Texas Health Science Center will serve as the main geographic recruitment site (although all recruitment and data collection take place online/via smartphone). All facilities and resources appear to be excellent for the conduct of the proposed project.
- The facilities/resources at the University of Washington appear to be well suited for accomplishing the aspects of the project to be addressed by those team members.

Weaknesses

- None noted.

Study Timeline:

Strengths

- The timeline, while not overly specific, seems to be consider timing needs relevant for each major study aims.

Weaknesses

- None noted.

Protections for Human Subjects:

Acceptable Risks and/or Adequate Protections

- A number of precautions are outlined to mitigate potential risks given the online nature of the proposed research.

Data and Safety Monitoring Plan (Applicable for Clinical Trials Only):

- Acceptable
    - o PI Lewis will be responsible for all data and safety oversight. A plan is described in the case of serious adverse events.

Inclusion Plans:

- Sex/Gender: Distribution justified scientifically
- Race/Ethnicity: Distribution justified scientifically
- For NIH-Defined Phase III trials, Plans for valid design and analysis:
- Inclusion/Exclusion Based on Age: Distribution justified scientifically
- Truncated age range is appropriate for the focus of the intervention. Characterization and considerations specific to gender minorities could be expanded upon more.

Resubmission:

- Very responsive to prior reviews

Resource Sharing Plans:

- Acceptable
- Not required but a data resource sharing plan is described.

Budget and Period of Support:

- Recommend as Requested

CRITIQUE 2

Significance: 2
Investigator(s): 2
Innovation: 3
Approach: 3

Environment: 2

Overall Impact:

This application seeks to examine motivations for using protective behavioral strategies (PBS) among young adult alcohol and cannabis users. The proposed research addresses an important public health problem. Substance abuse among young adults in the US is widespread and leads to a broad range of negative consequences. Addressing the ways to increase motivation to utilize protective behavioral strategies (PBS) is an important prevention goal. This application proposes to Use online focus groups to determine motivations for when, why, and how young adults may or may not use PBS when using alcohol and/or marijuana. Next, the investigators will conduct online and text message alcohol and marijuana PBS intervention among Concurrent Alcohol and Marijuana (CAM) use and Simultaneous Alcohol and Marijuana (SAM) at age 18-24. The proposed research, if carried out successfully, can facilitate the development of more efficacious interventions to reduce excessive alcohol use and marijuana use in young adults. The enthusiasm is slightly diminished by the lack of attention to potentially individualized nature of motivations to use PBS. Accounting for individual specific motivations could potentially increase the efficacy of the interventions.

1. Significance:

Strengths

- Alcohol and cannabis use among young adults is a major public health problem.
- There is little currently known about the reasons behind inconsistent use of protective behavioral strategies across different occasions.
- A better understanding of the factors driving the motivation for using protective behavioral strategies can potentially lead to more efficient prevention and intervention efforts.

Weaknesses

- None noted.

2. Investigator(s):

Strengths

- The Principal Investigator, Dr. Lewis, has substantial experience in the development and implementation of substance abuse interventions and is well qualified to conduct the research proposed in this application
- The collaborators in the team bring in additional expertise in other key areas of the study. Overall, the research team is well qualified to conduct the research proposed in this application.

Weaknesses

- None noted.

3. Innovation:

Strengths

- Available research on PBS related to marijuana use as well as concurrent and simultaneous marijuana and alcohol use is limited. The proposed study will bridge this important gap in knowledge.

Weaknesses

- Innovation is somewhat incremental because there has been previous research on PBS although it was mostly focused on alcohol phone.

4. Approach:

Strengths

- A two-stage approach in which the outcome of the first stage (Aim 1, motivations for using PBS) will inform the development and delivery of the intervention at the second stage (Aim 2) can potentially result in a more efficient intervention.

Weaknesses

- Motivations for using PBS may be different in different individuals in different contexts. The proposal gives little consideration to the potentially individualized nature of motivations, which may limit the efficacy of the interventions that take into account motivations to use PBS.

5. Environment:

Strengths

- The overall academic environment at the University of North Texas Health Science Center is excellent.
- The research facilities in the Studying Alcohol and Related Risks Lab are adequate for the execution of the proposed research.

Weaknesses

- None noted.

Study Timeline:

Strengths

- Timeline is appropriate

Weaknesses

- None noted

Protections for Human Subjects:

- Acceptable

Data and Safety Monitoring Plan (Applicable for Clinical Trials Only):

- Acceptable

Inclusion Plans:

- Sex/Gender: Distribution justified scientifically
- Race/Ethnicity: Distribution justified scientifically
- For NIH-Defined Phase III trials, Plans for valid design and analysis: Not applicable
- Inclusion/Exclusion Based on Age: Distribution justified scientifically
- acceptable

Resource Sharing Plans:

- Acceptable

Budget and Period of Support:

- Recommended budget modifications or possible overlap identified:

CRITIQUE 3

Significance: 1
Investigator(s): 1
Innovation: 1
Approach: 1
Environment: 1

Overall Impact:

This R34 from an exceptional team with complementarity expertise is aimed at developing prevention of cannabis and alcohol use problems in young adults using protective behavior strategies, with a particular focus on simultaneous or concurrent use of the two substances. More specifically, using focus groups and cognitive interviewing they will identify barriers to effective use of PBS by examining the whys/why-nots of using strategies, inconsistent or ineffective use of strategies, and interference from simultaneous use. Then they describe a pilot study (randomized trial with waitlist control) of a tailored program with a relevant sample of co-users with some readiness to change. The research is highly significant, programmatic, and well sequenced toward the next R01 study; the research is robust. The team was responsive to prior reviews, and this reviewer did not identify weaknesses in the application.

1. Significance:

Strengths

- Project will refine promising PBS models using methods that will generate concrete/actionable information on what weakens existing approaches.
- Addressing simultaneous and concurrent alcohol and cannabis use and increasing the knowledge base on cannabis PBS more generally is significant.
- Moves lit from focus on how to use PBS, to optimizing PBS interventions by addressing the why: “eliciting personally relevant reasons to make a change in one’s behavior and decide to use PBS’.

Weaknesses

- None noted.

2. Investigator(s):

Strengths

- Strong team of experts and consultants with experience in all aspects of this kind of research (PBS, EMA, complex longitudinal data analysis, retention, text messaging interventions and feedback, assessment of substance use and PBS, focus groups, clinical trials).
- Team members have collaborated previously on papers and grants.

- PI is highly accomplished, with expertise that extends beyond prevention of substance use to use-related consequences (e.g., sexual assault)—thus, they have a big picture perspective on the distal prevention implications of such work.
- Programmatic nature of the team's work is evident.

Weaknesses

- None noted.

3. Innovation:

Strengths

- Addresses simultaneous and concurrent cannabis and alcohol use.
- Addresses key neglected dimensions of PBS—e.g., the why and why not, quality/consistency of use
- Attention to inclusion of non-binary gender individuals

Weaknesses

- None noted.

4. Approach:

Strengths

- Waitlist control group
- Strong sequence of focus groups, cognitive interviewing, and program content development, and testing. Very well thought-out and described.
- Strong efforts to recruit young adults from a variety of community and online sources.
- Procedures are robust to pandemic related challenges.
- Additional procedures for creating non-binary focus groups when possible is a strength, and has potential for increasing impact, and for development of the next iterations of the program to be tested next (R01).
- Inclusion criteria aimed at generating a sample of individuals similar to who would be referred to such an intervention (high co-use, and some readiness to change).
- Some bio verification of substance use.

Weaknesses

- None noted

5. Environment:

Strengths

- Institutional support for research and intellectual environment is strong.
- Procedures are robust to pandemic related challenges.

Weaknesses

- None noted.

Study Timeline:

Strengths

- Approach to sequenced aims seems well thought out.

Weaknesses

- Details on the specific timing of various project activities was very general.

Protections for Human Subjects:

- addition of waitlist control is appropriate

Data and Safety Monitoring Plan (Applicable for Clinical Trials Only):

- well described

Inclusion Plans:

- Sex/Gender: Distribution justified scientifically
- Race/Ethnicity: Distribution not justified scientifically
- For NIH-Defined Phase III trials, Plans for valid design and analysis: Scientifically acceptable
- Inclusion/Exclusion Based on Age: Distribution justified scientifically
- well described

Resubmission:

- Responsive to prior reviews.

Budget and Period of Support:

- Recommend as Requested

THE FOLLOWING SECTIONS WERE PREPARED BY THE SCIENTIFIC REVIEW OFFICER TO SUMMARIZE THE OUTCOME OF DISCUSSIONS OF THE REVIEW COMMITTEE, OR REVIEWERS' WRITTEN CRITIQUES, ON THE FOLLOWING ISSUES:

PROTECTION OF HUMAN SUBJECTS: ACCEPTABLE

INCLUSION OF WOMEN PLAN: ACCEPTABLE

INCLUSION OF MINORITIES PLAN: ACCEPTABLE

INCLUSION ACROSS THE LIFESPAN: ACCEPTABLE

COMMITTEE BUDGET RECOMMENDATIONS: The budget was recommended as requested.

Footnotes for 1 R34 AA028730-01A1; PI Name: Lewis, Melissa A

NIH has modified its policy regarding the receipt of resubmissions (amended applications).See Guide Notice NOT-OD-18-197 at https://grants.nih.gov/grants/guide/notice-files/NOT-OD-18-197.html. The impact/priority score is calculated after discussion of an application by

averaging the overall scores (1-9) given by all voting reviewers on the committee and multiplying by 10. The criterion scores are submitted prior to the meeting by the individual reviewers assigned to an application, and are not discussed specifically at the review meeting or calculated into the overall impact score. Some applications also receive a percentile ranking. For details on the review process, see http://grants.nih.gov/grants/peer_review_process.htm#scoring.

MEETING ROSTER

Epidemiology, Prevention and Behavior Research Review Subcommittee
National Institute on Alcohol Abuse and Alcoholism Initial Review Group
NATIONAL INSTITUTE ON ALCOHOL ABUSE AND ALCOHOLISM
AA-2
10/19/2020 - 10/20/2020

Notice of NIH Policy to All Applicants: Meeting rosters are provided for information purposes only. Applicant investigators and institutional officials must not communicate directly with study section members about an application before or after the review. Failure to observe this policy will create a serious breach of integrity in the peer review process, and may lead to actions outlined in NOT-OD-14-073 at https://grants.nih.gov/grants/guide/notice-files/NOT-OD-14-073.html and NOT-OD-15-106 at https://grants.nih.gov/grants/guide/notice-files/NOT-OD-15-106.html, including removal of the application from immediate review.

CHAIRPERSON(S)

READ, JENNIFER P., PHD
PROFESSOR
DEPARTMENT OF PSYCHOLOGY
UNIVERSITY AT BUFFALO
THE STATE UNIVERSITY OF NEW YORK
BUFFALO, NY 14260

MEMBERS

ALLEN, JAMES R., PHD
PROFESSOR
DEPARTMENT OF BIOBEHAVIORAL HEALTH &
POPULATION SCIENCES
UNIVERSITY OF MINNESOTA MEDICAL SCHOOL, DULUTH
DULUTH , MN 55812

ANOKHIN, ANDREY P., PHD *
PROFESSOR
DEPARTMENT OF PSYCHIATRY
SCHOOL OF MEDICINE
WASHINGTON UNIVERSITY
ST. LOUIS, MO 63110

CAETANO, RAUL, PHD, MD, MPH
SENIOR RESEARCH SCIENTIST
PREVENTION RESEARCH CENTER
PACIFIC INSTITUTE FOR RESEARCH AND EVALUATION
OAKLAND, CA 94612

FAIRBAIRN, CATHARINE, PHD *
ASSISTANT PROFESSOR
DEPARTMENT OF PSYCHOLOGY
UNIVERSITY OF ILLINOIS AT URBANA-CHAMPAIGN
CHAMPAIGN, IL 61820

FURR-HOLDEN, C. DEBRA M, BA, PHD
C.S. MOTT ENDOWED PROFESSOR OF PUBLIC HEALTH
PROFESSOR
DEPARTMENT OF EPIDEMIOLOGY AND BIOSTATISTICS
COLLEGE OF HUMAN MEDICINE
MICHIGAN STATE UNIVERSITY
FLINT, MI 48502

GIZER, IAN ROBERT, PHD
ASSOCIATE PROFESSOR
DEPARTMENT OF PSYCHOLOGICAL SCIENCES
UNIVERSITY OF MISSOURI, COLUMBIA
COLUMBIA, MO 65211

HOUSTON, REBECCA J, PHD *
ASSISTANT PROFESSOR
HEALTH AND ADDICTIONS RESEARCH CENTER
DEPARTMENT OF PSYCHOLOGY
ROCHESTER INSTITUTE OF TECHNOLOGY
ROCHESTER, NY 14623

KERR, DAVID C. R., PHD
ASSOCIATE PROFESSOR
DEPARTMENT OF PSYCHOLOGY
SCHOOL OF PSYCHOLOGICAL SCIENCE
OREGON STATE UNIVERSITY
CORVALLIS, OR 97331

KEYES, KATHERINE MARGARET, PHD
ASSOCIATE PROFESSOR
DEPARTMENT OF EPIDEMIOLOGY
MAILMAN SCHOOL OF PUBLIC HEALTH
COLUMBIA UNIVERSITY
NEW YORK, NY 10032

MCGUE, MATTHEW K., PHD
PROFESSOR
DEPARTMENT OF PSYCHOLOGY
MEMBER, INSTITUTE OF HUMAN GENETICS
UNIVERSITY OF MINNESOTA
MINNEAPOLIS, MN 55455

ONDERSMA, STEVEN J, PHD *
PROFESSOR
DEPARTMENT OF PSYCHIATRY & BEHAVIORAL NEUROSCIENCE
AND OBSTETRICS & GYNECOLOGY
MERRILL PALMER SKILLMAN INSTITUTE
WAYNE STATE UNIVERSITY SCHOOL OF MEDICINE
DETROIT, MI 48236

PASCHALL, MALLIE J, PHD *
SENIOR RESEARCH SCIENTIST
PREVENTION RESEARCH CENTER
OAKLAND, CA 94612

PATRICK, MEGAN ELIZABETH, PHD
RESEARCH PROFESSOR
INSTITUTE FOR SOCIAL RESEARCH
UNIVERSITY OF MICHIGAN
ANN ARBOR, MI 48109

REID, ALLECIA E., AB, MA, PHD *
ASSISTANT PROFESSOR
ASSISTANT PROFESSOR
DEPARTMENT OF PSYCHOLOGICAL AND BRAIN SCIENCES
UNIVERSITY OF MASSACHUSETTS
AMHERST, MA 01003

ROMANO, EDUARDO O, PHD *
SENIOR RESEARCH SCIENTIST
PACIFIC INSTITUTE FOR RESEARCH AND EVALUATION
11720 BELTSVILLE DR., SUITE 900
CALVERTON, MD 20705

SANCHEZ, MARIANA, MSW, PHD *
ASSISTANT PROFESSOR
DEPARTMENT OF HEALTH PROMOTION AND DISEASE PREVENTION
FLORIDA INTERNATIONAL UNIVERSITY
MIAMI, FL 33199

SCIENTIFIC REVIEW OFFICER

GHAMBARYAN, ANNA, MD, PHD
SCIENTIFIC REVIEW OFFICER
EXTRAMURAL PROJECT REVIEW BRANCH
OFFICE OF EXTRAMURAL ACTIVITIES
NATIONAL INSTITUTE ON ALCOHOL ABUSE AND ALCOHOLISM
NATIONAL INSTITUTES OF HEALTH
BETHESDA, MD 20892

EXTRAMURAL SUPPORT ASSISTANT

FULTON, THELMA
EXTRAMURAL SUPPORT ASSISTANT
OFFICE OF EXTRAMURAL ACTIVITIES
NATIONAL INSTITUTE ON ALCOHOL ABUSE AND ALCOHOLISM
NATIONAL INSTITUTES OF HEALTH
ROCKVILLE, MD 20892-9304

* Temporary Member. For grant applications, temporary members may participate in the entire meeting or may review only selected applications as needed.

Consultants are required to absent themselves from the room during the review of any application if their presence would constitute or appear to constitute a conflict of interest.
